# Incorporating Tissue-Specific Gene Expression Data to Improve Chemical–Disease Inference of in Silico Toxicogenomics Methods

**DOI:** 10.3390/jox14030057

**Published:** 2024-07-31

**Authors:** Shan-Shan Wang, Chia-Chi Wang, Chien-Lun Wang, Ying-Chi Lin, Chun-Wei Tung

**Affiliations:** 1 Ph.D. Program in Environmental and Occupational Medicine, College of Medicine, Kaohsiung Medical University and National Health Research Institutes, Kaohsiung 80708, Taiwan; shanwang@nhri.edu.tw; 2 Institute of Biotechnology and Pharmaceutical Research, National Health Research Institutes, Miaoli County 35053, Taiwan; 3 Graduate Institute of Data Science, College of Management, Taipei Medical University, Taipei 10675, Taiwan; i906108002@tmu.edu.tw; 4 Department and Graduate Institute of Veterinary Medicine, School of Veterinary Medicine, National Taiwan University, Taipei 10617, Taiwan; ccwang@ntu.edu.tw; 5 Master and Doctoral Degree Program in Toxicology, College of Pharmacy, Kaohsiung Medical University, Kaohsiung 80756, Taiwan; yclin@kmu.edu.tw; 6 School of Pharmacy, College of Pharmacy, Kaohsiung Medical University, Kaohsiung 80756, Taiwan

**Keywords:** in silico toxicogenomics, tissue-specific gene expression, tissue-specific protein expression, chemical–disease inference, enrichment analysis

## Abstract

In silico toxicogenomics methods are resource- and time-efficient approaches for inferring chemical–protein–disease associations with potential mechanism information for exploring toxicological effects. However, current in silico toxicogenomics systems make inferences based on only chemical–protein interactions without considering tissue-specific gene/protein expressions. As a result, inferred diseases could be overpredicted with false positives. In this work, six tissue-specific expression datasets of genes and proteins were collected from the Expression Atlas. Genes were then categorized into high, medium, and low expression levels in a tissue- and dataset-specific manner. Subsequently, the tissue-specific expression datasets were incorporated into the chemical–protein–disease inference process of our ChemDIS system by filtering out relatively low-expressed genes. By incorporating tissue-specific gene/protein expression data, the enrichment rate for chemical–disease inference was largely improved with up to 62.26% improvement. A case study of melamine showed the ability of the proposed method to identify more specific disease terms that are consistent with the literature. A user-friendly user interface was implemented in the ChemDIS system. The methodology is expected to be useful for chemical–disease inference and can be implemented for other in silico toxicogenomics tools.

## 1. Introduction

Chemical safety is important for human health, since the exposure to harmful chemicals can lead to adverse effects. Current chemical hazard assessment is moving from traditional animal models to non-animal alternatives that not only comply with the animal 3Rs (Replacement, Reduction, and Refinement) principle but are also expected to mitigate bias from species differences. Among the non-animal alternatives, toxicogenomics (TGx) is one of the promising tools that utilizes genomics and bioinformatics techniques to study adverse outcomes [1]. For example, gene biomarkers for specific toxicity can be identified using TGx techniques. The identified gene biomarkers can be utilized for predicting potential toxicants and providing a better understanding of underlying mechanisms [2,3]. TGx techniques also facilitate the development of adverse outcome pathways (AOPs) for chemical toxicity assessment [4].

In silico TGx is a special type of TGx method that utilizes a database of chemical-gene/protein interactions instead of conducting transcriptomics experiments. The ChemDIS system [5,6] and the Comparative Toxicogenomics Database (CTD) [7,8] are two representative online systems for inferring affected diseases via analyzing chemical–gene/protein–disease associations. One of their major differences is the chemical–gene/protein interaction database applied for inference. A manually curated database of chemical–gene/protein interactions was utilized in CTD [9], while ChemDIS utilized the STITCH database [10], which is the largest chemical–protein interaction database. In silico TGx methods are also helpful for AOP development [11,12] and can be utilized as complementary evidence for weight-of-evidence assessment of chemical toxicity [13,14].

Despite the usefulness of the in silico TGx methods, the utilized interaction data are obtained either from the literature or predicted interactions without considering tissue-specific gene expression. Since many genes are preferentially expressed in specific tissues [15,16], the conventional disease inference process could result in many false positives by considering all genes without filtering out low-expressed genes in the corresponding disease-relevant tissues. In this study, six tissue-specific gene expression data consisting of four gene expression and two protein expression data were extracted from Expression Atlas [17] and analyzed to define three expression levels that can be utilized for filtering out genes with relatively low expression levels. The tissue-specific expression data and defined expression levels were then incorporated into the ChemDIS inference system to demonstrate its usefulness in improving chemical–disease inference. The results showed that the exclusion of low-expression genes can provide up to 62.26% improvement in the enrichment rate, which is the percentage of enriched chemical–disease associations that were curated by the CTD database. The function was implemented as an option to be included in the online ChemDIS system to facilitate the chemical–disease inference. As a case study, an analysis of melamine was conducted to compare in silico TGx analyses with and without tissue-specific gene expression information. The incorporation of tissue-specific gene expression information is expected to be also useful for other in silico TGx methods.

## 2. Materials and Methods

### 2.1. Gene/Protein Expression Dataset

Gene and protein expression datasets tested in more than 15 human tissues were downloaded from EMBL-EBI Expression Atlas [17]. In this study, we focused on the expression data in human tissues to match the analysis functions of ChemDIS. As a result, four RNA sequencing-based gene expression datasets, one quantitative proteomics datum, and one semi-quantitative proteomics datum were collected for the following analysis. The number of studied tissues ranged from 16 to 53, and the number of genes ranged from 43,723 to 46,754 for the four gene expression datasets. The expression value for gene expression datasets was represented as transcript per million (TPM) and ranged from 0 to 315,499. The quantitative proteomics dataset comprised 31 tissues and 12,832 proteins with expression values ranging from 0 to 183,223,473. The semi-quantitative proteomics dataset included 44 tissues and 5109 proteins with expression values categorized into three levels: low, medium, and high. The expression values in the proteomics dataset were represented as parts per billion (ppb). The summary of each dataset is listed in Table 1, and the whole list of tissues included in each dataset is shown in Table S1.

### 2.2. Thresholds for Defining Low, Medium, and High Expression Levels

**Figure 1:**
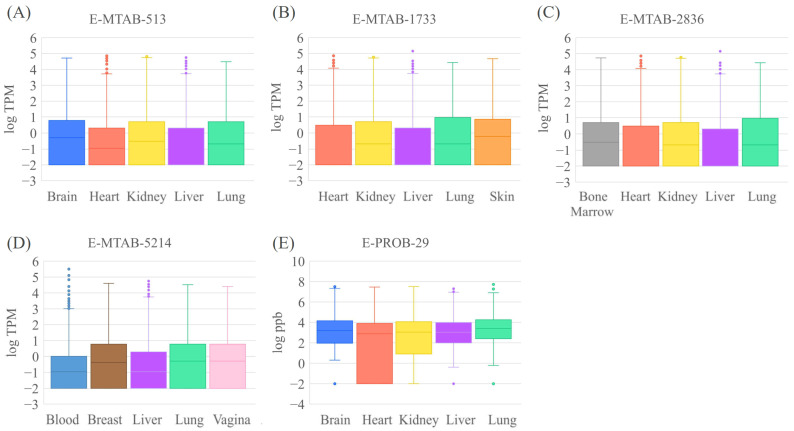
Boxplot of the expression value for the tissue-specific gene expression datasets of (**A**) E-MTAB-513, (**B**) E-MTAB-1733, (**C**) E-MTAB-2836, (**D**) E-MTAB-5214, (**E**) E-PROB-29.

Due to the large variation in gene expression values and no common criteria for classifying gene expression levels, we conducted analyses on the distribution of gene/protein expression values. For the four RNA-seq datasets, the 25th percentiles of TPM expression for all ten tissues of blood, skin, brain, lung, heart, kidney, liver, breast, bone marrow and vagina are zero. The 50th percentiles of TPM expression for the ten tissues ranged from 0 to 0.6. The 75th percentiles of TPM expression for the ten tissues ranged from 1 to 9, which means that at least 75% of the gene expression values in this dataset are less than 9. The distribution of gene expression values represented as logTPM is shown in Figure 1. Please note that a small number of 0.01 was added to the TPM values to avoid an invalid logarithm of zero. Three thresholds for filtering out low-, medium- and high-expression genes were set to 0.5, 1, and 5, representing average percentiles of 57, 63, and 75, respectively. A gene with expression values less than or equal to 0.5 was considered to be a low-expression gene, while a gene with an expression value greater than 5 was considered to have a very high expression value. For the quantitative proteomics dataset, we set threshold values of 500, 1000, and 1500 (ppb) to represent the low, medium and high gene expression levels, respectively. Three thresholds for filtering out low-, medium- and high-expression genes were set to 500, 1000, and 1500, representing average percentiles of 39, 47, and 52, respectively. Please note that the expression levels of E-PROT 3 was taken from its original dataset, which is a semi-quantitative analysis with predefined low, medium, and high expression levels.

### 2.3. Methodology of Chemical–Protein–Disease Inference

For chemical–protein–disease inference, we utilized the ChemDIS system [5,6]. The ChemDIS system is capable of analyzing more than 430,000 chemicals based mainly on the 15 million chemical–protein interactions extracted from the STITCH 5 database [10]. The webserver is freely available at https://cwtung.nhri.edu.tw/chemdis (accessed on 25 April 2024). The inference process includes three steps. First, chemical-interacting proteins were identified with associated disease ontology (DO) terms [27,28]. Second, for each DO term, hypergeometric tests with Benjamini–Hochberg correction for multiple testing were conducted to calculate the adjusted *p*-value. Third, DO terms with an adjusted *p*-value less than 0.05 were identified to be the inferred diseases affected by a given chemical.

**Figure 2:**
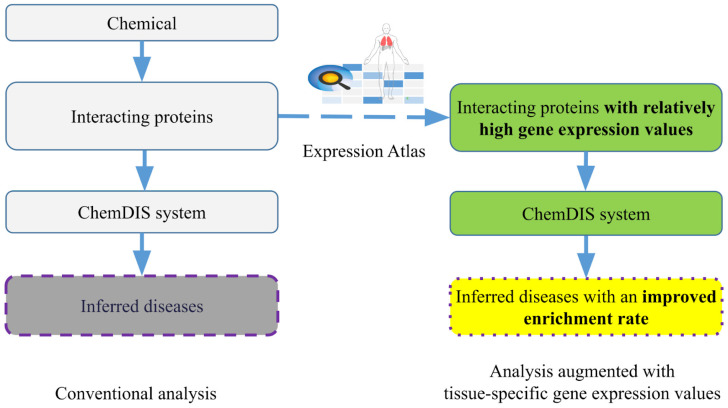
The system flows for the conventional and augmented methods.

Compared to conventional methods considering all interacting proteins, the proposed method incorporates tissue-specific gene expression data to augment disease inference. The incorporation of tissue-specific gene expression information adds an additional step for filtering out the interacting proteins with relatively low expression identified in the above-mentioned first step. Specifically, the thresholds for low, medium, and high expression levels defined in the previous section were applied to filter out interacting proteins with relatively low expression levels. The remaining proteins were then utilized for enrichment analysis. In this way, the enrichment analysis will be conducted using only proteins with relatively abundant gene expression levels. Figure 2 shows the system flow of the two methods.

### 2.4. Dataset and Measurement for Evaluating Model Performance

Curated evidence of chemical–disease associations was retrieved from CTD on 7 October 2021 and was utilized for evaluating the inference performance of ChemDIS with and without tissue-specific gene expression data. The CTD database provides a comprehensive knowledge base of high-quality associations of chemical and disease that are suitable for evaluating the proposed method. The tissue–disease associations were identified by keyword searches using the web user interface of the Disease Ontology (DO) database at https://disease-ontology.org/do (accessed on 7 October 2021) [27,28] and text matching using the obo file obtained from https://obofoundry.org/ontology/doid.html (accessed on 7 October 2021). The keywords were the organ names provided by the datasets shown in Table 2. Since some tissues were associated with only a few DO terms, the performance measurement on these tissues could be biased. To mitigate the bias, we selected the top five tissues that were associated with the highest number of diseases in each dataset. Table 2 shows the five tissues for each dataset. A total of ten tissues were considered in the performance evaluation. A total of 128, 98, 73, 62, 50, 37, 31, 22, 22, and 18 DO terms are associated with blood, skin, brain, lung, heart, kidney, liver, breast, bone marrow, and vagina. There are 3935 chemicals and 431 chemical–disease associations for performance evaluation in this study. Please note that there are still many undiscovered chemical–disease associations; therefore, some conventional performance measurements such as accuracy and specificity may not be suitable. Given *x* inferred associations and *y* CTD curated associations, the discovery rate and enrichment rate were utilized in this study as defined in the following equations.

Discovery rate = (*x* ∩ *y*)/*y* (1)

Enrichment rate = (*x* ∩ *y*)/*x* (2)

All the analyses were implemented using R programming language and packages dplyr to process the data [29].

## 3. Results and Discussion

### 3.1. Diseases Inference Augmented by Incorporating Tissue-Specific Gene Expression

**Figure 3:**
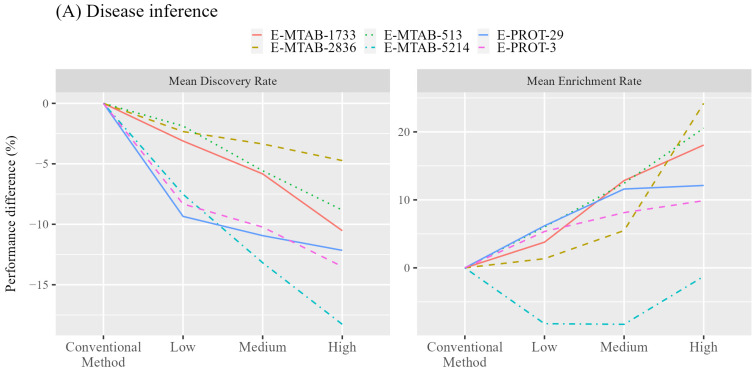

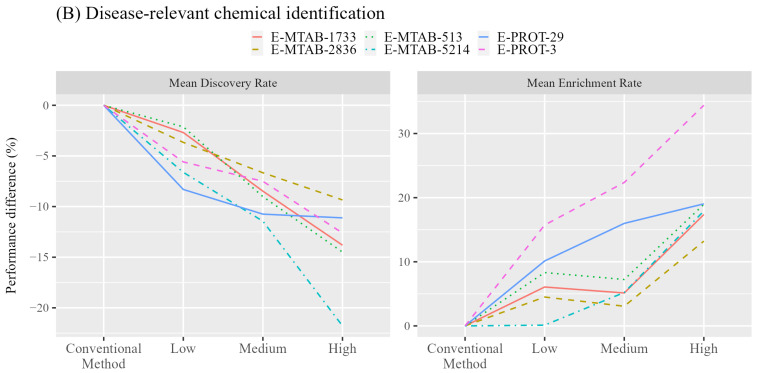
Performance comparison of conventional and augmented methods using three expression filters for (**A**) disease inference and (**B**) disease-relevant chemical identification.

A total of 3935 chemicals annotated to be associated with the diseases of the 10 tissues were analyzed by conventional and augmented methods. Figure 3A shows the average discovery and enrichment rates for the evaluated chemicals. Detailed performance for each tissue is available in Figures S1–S6. As expected, the average discovery rate was decreased in all evaluated tissues when excluding the low-expression genes. Liver is the only tissue with only a minor decreased discovery rate (−7.29%), where the filtering of low-expression genes is beneficial with 4.38%, 4.30%, 1.47%, and 0.59% improvement on the discovery rate for RNA-seq datasets of E-MTAB-1733, E-MTAB-2836, E-MTAB-513, and E-MTAB-5214 but not for proteomics datasets. In contrast, a large improvement in the average enrichment rate was obtained by filtering out low-expression genes for six tissues of bone marrow, brain, heart, kidney, liver, and skin with 5.75%, 6.68%, 6.84%, 6.46%, 13.33%, and 8.62% improvement, respectively. The filtering using all datasets provided an improved enrichment rate except for E-MTAB-5214, where improvement was observed only for the liver. The improvement in enrichment rate increases when applying a stringent filtering threshold, as shown in Figure 3A.

The highest enrichment rates were achieved by applying the filter of high-expression level, i.e., only genes/proteins with very high expression values were utilized for enrichment analysis. Compared to the conventional method, the three tissues with the highest improvement in average enrichment rate are the heart, liver, and kidney with 34.21%, 24.65%, and 19.93% improvement, respectively. The use of the heart-specific expression data of E-MTAB-2836 yielded the highest improvement of 44.79% in the enrichment rate. Please note that this study considered only the annotated DO terms obtained from the CTD database. Since there could still be undiscovered chemical–disease associations, the enrichment rate could be underestimated. As the curation process may prefer a higher level DO term representing a general concept rather than a specific one, the augmented analysis with fewer interacting proteins may tend to predict DO terms of lower levels for more specific diseases and miss the DO terms consisting of a high number of involved proteins. In this aspect, in addition to the enrichment rate improvement, the benefit obtained from the augmented analysis for identifying more specific DO terms is considered more useful for analyzing chemical–disease associations.

### 3.2. Identification of Disease-Relevant Chemicals

Since the disease inference for a given chemical was successfully improved by incorporating tissue-specific gene expression data, it would be interesting to know whether chemicals associated with a specific disease can be identified for hazard chemical identification. We therefore conducted enrichment analysis for 344,471 chemicals whose inference yielded at least one disease in the ChemDIS system using conventional and augmented methods and compared the results. The diseases associated with the same 10 tissues were included in this analysis and discovery, and enrichment rates were calculated for comparison. The average performance improvements for the six datasets are shown in Figure 3B. Detailed performance for each tissue and the tissue-expression dataset is available in Figures S7–S12.

Similarly, a decreased average discovery rate and increased enrichment rate were obtained by applying a more stringent tissue-specific expression filter. While a negative effect on the performance was observed for the disease inference using the E-MTAB-5213, performance improvement was observed for the identification of disease-relevant chemicals. For applying the low-expression filter, the three tissues with the highest improvement in average enrichment rate are the heart, skin, and blood with 17.89%, 13.13%, and 10.57% improvement, respectively. Among the three tissues, the average discovery rates for heart and blood decreased with 3.57% and 15.41% lower performance, respectively, while a 2.97% improvement was obtained for the skin. Only the brain and vagina were associated with a lower average enrichment rate using the low-expression filter. The application of the high-expression filter resulted in a more significant improvement in the enrichment rate, where the vagina is the only tissue without improvement. The three tissues with the highest improvement in average enrichment rate are the blood, heart, and skin with 47.57%, 45.24%, and 36.07% improvement, respectively. The use of the lung-specific expression data of E-PROT-3 for analyzing disease-relevant chemicals yielded the highest improvement of 62.26% in the enrichment rate. Generally, the incorporation of tissue-specific gene expression data can benefit the identification of disease-relevant chemicals.

### 3.3. Web-Based User Interface

**Figure 4:**
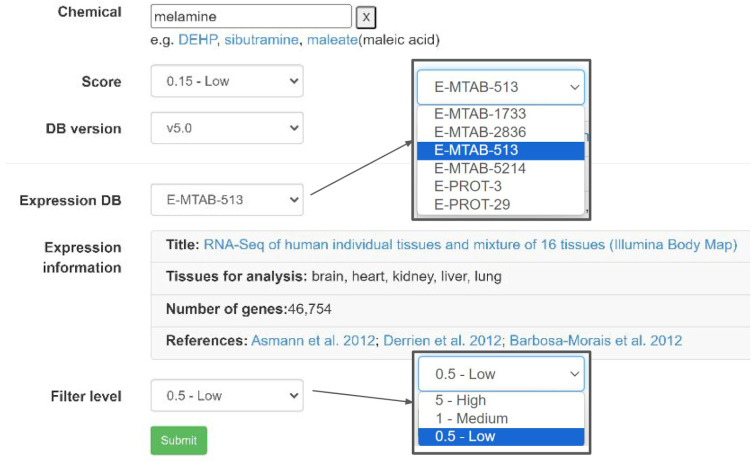
The user interface of ChemDIS with augmented functions for incorporating tissue-specific expression data. Users can select the expression dataset (E-MTAB-513 [18,19,20] in this example) and filter level for analysis.

The datasets and analysis workflow have been implemented in the ChemDIS platform using Angular, Golang, and MongoDB and are available at https://cwtung.nhri.edu.tw/chemdis (accessed on 25 April 2024). As shown in Figure 4, three essential input fields of the query chemical, confidence threshold of interacting proteins, and database version should be entered in the same way as the previous system. The tissue-specific filters were implemented as two additional dropdown menus for the tissue-specific dataset and level for filtering out relatively low expressed genes. Once a dataset is selected, the corresponding information of the dataset will be shown as a table including the title, available tissues for analysis, number of genes included in the dataset, and the references.

**Figure 5:**
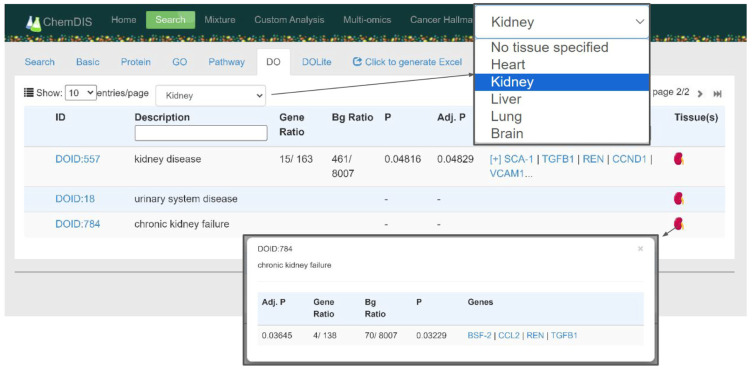
The improved disease ontology (DO) analysis results. There is a new dropdown menu for showing only the DO terms associated with the tissue of interest and a new tissue column showing the results of enrichment analysis augmented by incorporating the tissue-specific expression data, respectively.

Figure 5 shows an example of the analysis results of melamine. In the disease ontology (DO) tab, the original layout of the ChemDIS system was kept with enriched DO term ID, description, gene ratio, background ratio, *p*-value, adjusted *p*-value, and interacting genes belonging to the DO term. Two new items of a dropdown menu were for showing only the DO terms associated with specified tissue expression data and a new column showing the results of the incorporation of tissue-specific expression data, respectively. As a default, there will be no tissue specified in the filtering dropdown menu, and all results will be shown in the table. A color icon and gray icon of a tissue indicate enrichment and no enrichment by incorporating tissue-specific expression data, respectively. By clicking the kidney icon with color, the enrichment analysis results will pop up showing the *p*-value, adjusted *p*-value, gene ratio, background ratio, and involved genes. In this case, the urinary system disease and chronic kidney failure were not enriched in the original ChemDIS system but were enriched by filtering out low-expression genes, which will be further discussed in the next section.

### 3.4. Case Study: Melamine

To demonstrate the usefulness of the augmented analysis method by incorporating tissue-specific gene expression data, a well-studied food contact chemical melamine with a relatively comprehensive interacting gene/protein profile and known affected diseases was chosen. Melamine is a nephrotoxic compound that can migrate from food contact material. Exposure to melamine was found to increase the risk of urinary system diseases [30]. In addition, the association between melamine and central nervous system disease was reported in previous studies [31,32]. To show the benefit of the augmented analysis, conventional and augmented methods were utilized to analyze melamine, and their results were compared. Based on the previously reported effects, this analysis focused on brain and kidney-relevant diseases. E-MTAB-513 and E-PROT-29 were utilized for brain disease inference, while all datasets were utilized for kidney disease inference except for E-MTAB-5214, as shown in Table 2. Three levels of filters were utilized for analysis, and their inference results are available in Table S2.

**Figure 6:**
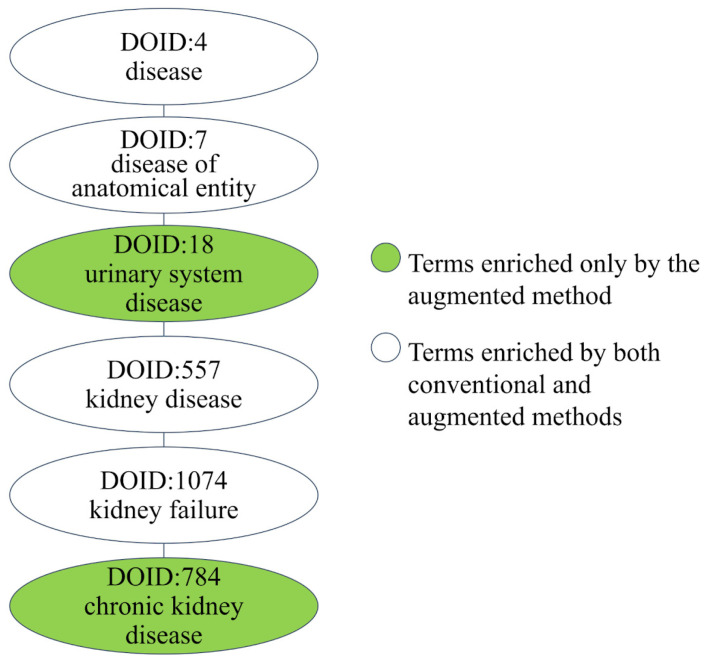
The disease term hierarchy of chronic kidney failure enriched by augmented and conventional methods.

A total of 316 interacting proteins of melamine were identified in the conventional method using the STITCH v5 database. In comparison, the application of the low-expression filter using brain expression data from E-MTAB-5 and E-ROPT-29 resulted in 262 and 241 interacting proteins included in the augmented analysis, respectively. As for the kidney, a total of 257, 259, 254, 280, and 239 interacting proteins for E-MTAB-1733, E-MTAB-2836, E-MTAB-513, E-PROT-3, and E-PROT-29 were included in the augmented analysis by removing low-expression genes. Figure 6 shows the hierarchy of chronic kidney failure inferred by the conventional method and augmented method using the low-expression filter of E-MTAB-513, where the conventional method inferred only partial nodes of the hierarchy, but the augmented method inferred the complete hierarchy. Both methods are able to infer the high-level DO terms, but the more specific term of chronic kidney failure was only inferred by the augmented method. The identification of chronic kidney failure (DOID: 784) is consistent with previous studies [33,34]. In the conventional method, 11 out of 365 (3.01%) inferred DO terms were kidney-relevant terms. In contrast, the augmented analysis based on E-MTAB-513 showed a higher percentage of DO terms relevant to the kidney (13/305 = 4.26%).

Another example is the use of E-PROT-29 for filtering out low- and medium-expression proteins in the brain; the results showed that 6.32% (18/285) of the inferred DO terms were associated with brain diseases compared to 3.84% (14/365) using the conventional method. In the conventional method, central nervous system disease (DOID: 331) and its child node of brain disease (DOID: 936) were identified. In contrast, a more specific term of movement disease (DOID: 480), which is a child node of brain disease, was identified by the augmented method. A recent study also suggests the potential link of melamine consumption to movement diseases such as Parkinson’s disease [35]. The more specific terms inferred by the augmented are more useful for hazard identification.

## 4. Conclusions

In silico toxicogenomics methods are powerful tools for establishing experimental hypotheses and can be utilized as complementary evidence for predicting toxicities with complex mechanisms. However, the inclusion of low-expression genes in the disease inference can generate biased results. As tissue-specific effects of xenobiotics exist [36,37,38,39] and tissue-specific transcriptomics analysis can reveal the potential mechanisms of xenobiotics [40,41,42,43], in silico toxicogenomics analysis capable of incorporating tissue-specific expression is considered more useful for revealing the tissue-specific effects. The present work analyzed six tissue-specific expression datasets and incorporated them into the disease inference process by defining three thresholds for filtering out relatively low-expression genes. The results showed a large improvement in the enrichment rate but a decreased discovery rate that is reasonable for reducing gene numbers for analysis. The case study of melamine showed that the incorporation of tissue-specific expression data can both improve the enrichment rate and identify more specific disease terms that are considered more useful for hazard identification and experimental validation. An updated web server has been implemented to incorporate the tissue-specific expression filter in the ChemDIS system.

The analysis of transcriptomics data can facilitate the development of AOP for a specific toxicity. However, the transcriptomics analysis requires extensive experiments. The developed augmented in silico toxicogenomics method is considered to be beneficial for that purpose compared to conventional methods. The common interacting proteins of chemicals corresponding to a specific toxicity can be identified to be the candidate molecular initiating events using the proposed method, and the common inferred functions and pathways can be identified as candidate key events. The augmented method is expected to reduce false positives by filtering out relatively low-expressed genes/proteins.

While upregulation and downregulation of the genes associated with chemical exposure could be critical for determining potential toxic or therapeutic effects, in silico toxicogenomics systems are currently not able to consider the direction of regulation. The major issue for incorporating the idea is the lack of data sources on gene regulation by chemical exposure. Some considerations should be taken into account. For example, the direction of gene regulation can be condition-dependent, but current in silico toxicogenomics methods consider only the average condition. In addition, the directions of genes could be opposite, resulting in no consensus conclusion of a toxic effect or a therapeutic effect.

While only five tissues for each dataset were utilized to evaluate the model performance, the expression data of the other tissues of the datasets are expected to be also useful for tissue-specific analysis of chemical–disease inference. Future works include the implementation of analysis functions for all tissues provided by the datasets, the incorporation of more tissue-specific expression data to further extend the tissue-specific analysis function, and the development of a methodology for developing an AOP hypothesis using the proposed method. The methodology proposed in this study is also expected to be useful for other in silico toxicogenomics systems. For example, in silico toxicogenomics tools for cross-species analysis can leverage tissue-specific expression information for different species to develop expression-level filters for different species and study the potential species-dependent effects of chemical exposure instead of considering all interacting genes collected from all species that could produce incorrect inference.

## Figures and Tables

**Table 1:** Summary of the gene/protein expression datasets.

Type	ID	Tissues	Expression Value	References
RNA-Seq mRNA	E-MTAB-513	16	0 to 75,295 TPM	[18,19,20]
	E-MTAB-5214	53	0 to 315,499 TPM	[21]
	E-MTAB-2836	32	0 to 137,860 TPM	[22,23]
	E-MTAB-1733	27	0 to 137,868 TPM	[24]
Proteomics	E-PROT-3	44	1 (low), 2 (medium), 3 (high)	[22,25]
	E-PROT-29	31	0 to 51,851,041 ppb	[26]

TPM: transcript per million; ppb: parts per billion.

**Table 2:** The five tissues with the highest number of annotated diseases from CTD for each dataset.

ID	Blood (*n* = 128)	Skin (*n* = 98)	Brain (*n* = 73)	Lung (*n* = 62)	Heart (*n* = 50)	Kidney (*n* = 37)	Liver (*n* = 31)	Breast (*n* = 22)	Bone Marrow (*n* = 22)	Vagina (*n* = 18)
E-MTAB-513			V	V	V	V	V			
E-MTAB-5214	V			V			V	V		V
E-MTAB-2836				V	V	V	V		V	
E-MTAB-1733		V		V	V	V	V			
E-PROT-3				V		V	V	V	V	
E-PROT-29			V	V	V	V	V			

V: the tissue was among the top five tissues with the highest number of annotated diseases from CTD.

## Data Availability

Data are available at Expression Atlas and the ChemDIS server is publicly accessible at https://cwtung.nhri.edu.tw/chemdis (accessed on 25 April 2024).

## Supplementary Materials

The following supporting information can be downloaded at https://www.mdpi.com/article/10.3390/jox14030057/s1, Figure S1: The performance of conventional disease inference and inference by incorporating tissue expression filters for the E-MTAB-5214 dataset; Figure S2: The performance of conventional disease inference and inference by incorporating tissue expression filters for the E-MTAB-513 dataset; Figure S3: The performance of conventional disease inference and inference by incorporating tissue expression filters for the E-MTAB-1733 dataset; Figure S4: The performance of conventional disease inference and inference by incorporating tissue expression filters for the E-MTAB-2836 dataset; Figure S5: The performance of conventional disease inference and inference by incorporating tissue expression filters for the E-PROT-29 dataset; Figure S6: The performance of conventional disease inference and inference by incorporating tissue expression filters for the E-PROT-3 dataset; Figure S7: The performance for identifying disease-relevant chemicals of the conventional method and augmented methods by incorporating tissue expression filters for the E-MTAB-5214 dataset; Figure S8: The performance for identifying disease-relevant chemicals of the conventional method and augmented methods by incorporating tissue expression filters for the E-MTAB-513 dataset; Figure S9: The performance for identifying disease-relevant chemicals of the conventional method and augmented methods by incorporating tissue expression filters for the E-MTAB-1733 dataset; Figure S10: The performance for identifying disease-relevant chemicals of the conventional method and augmented methods by incorporating tissue expression filters for the E-MTAB-2836 dataset; Figure S11: The performance for identifying disease-relevant chemicals of the conventional method and augmented methods by incorporating tissue expression filters for the E-PORT-29 dataset; Figure S12: The performance for identifying disease-relevant chemicals of the conventional method and augmented methods by incorporating tissue expression filters for the E-PORT-3 dataset; Table S1: Lists of tissues for the gene/protein expression datasets; Table S2: Selected analysis results of melamine based on conventional and augmented methods.
