# Supplementary material for: Incorporating Tissue-Specific Gene Expression Data to Improve Chemical–Disease Inference of in Silico Toxicogenomics Methods
Source: J Xenobiot. 2024 Jul 31;14(3):1023–35. doi: 10.3390/jox14030057 (PMC11348041; doi:10.3390/jox14030057)
Supplement: Supplementary file 1 [file jox-14-00057-s001.zip › FigureS.pdf]

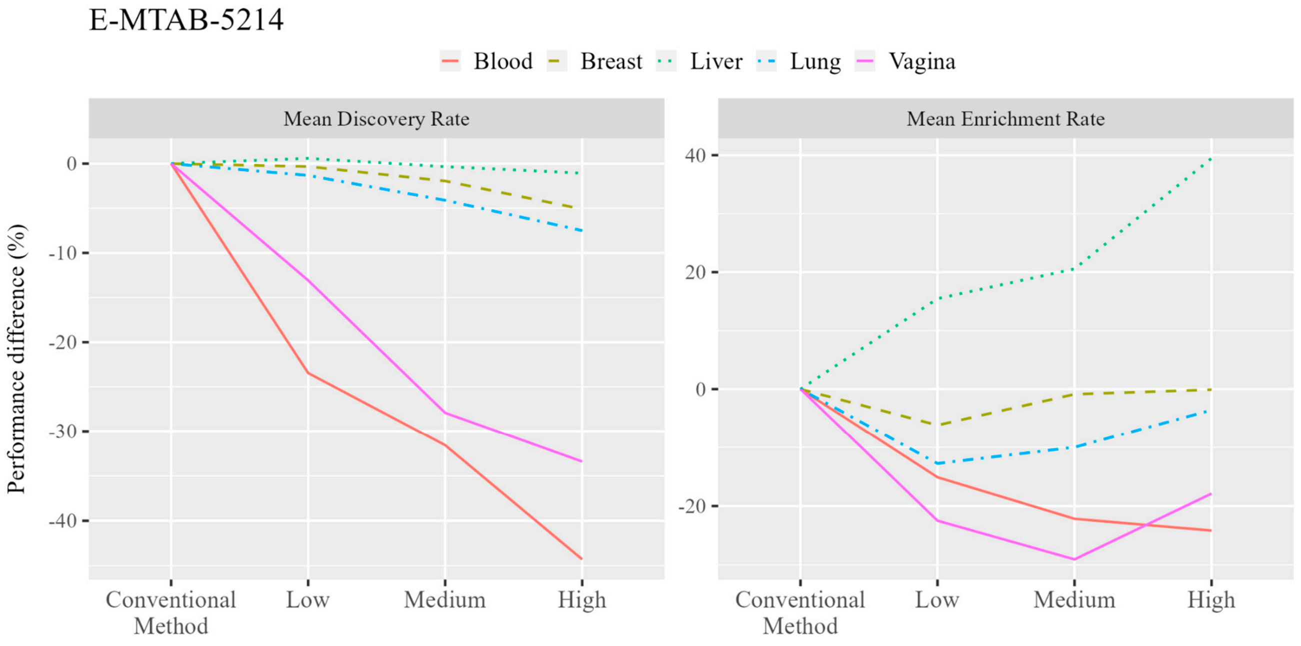

Figure S1. The performance of conventional disease inference and inference by incorporating tissue expression filters for the E-MTAB-5214 dataset.

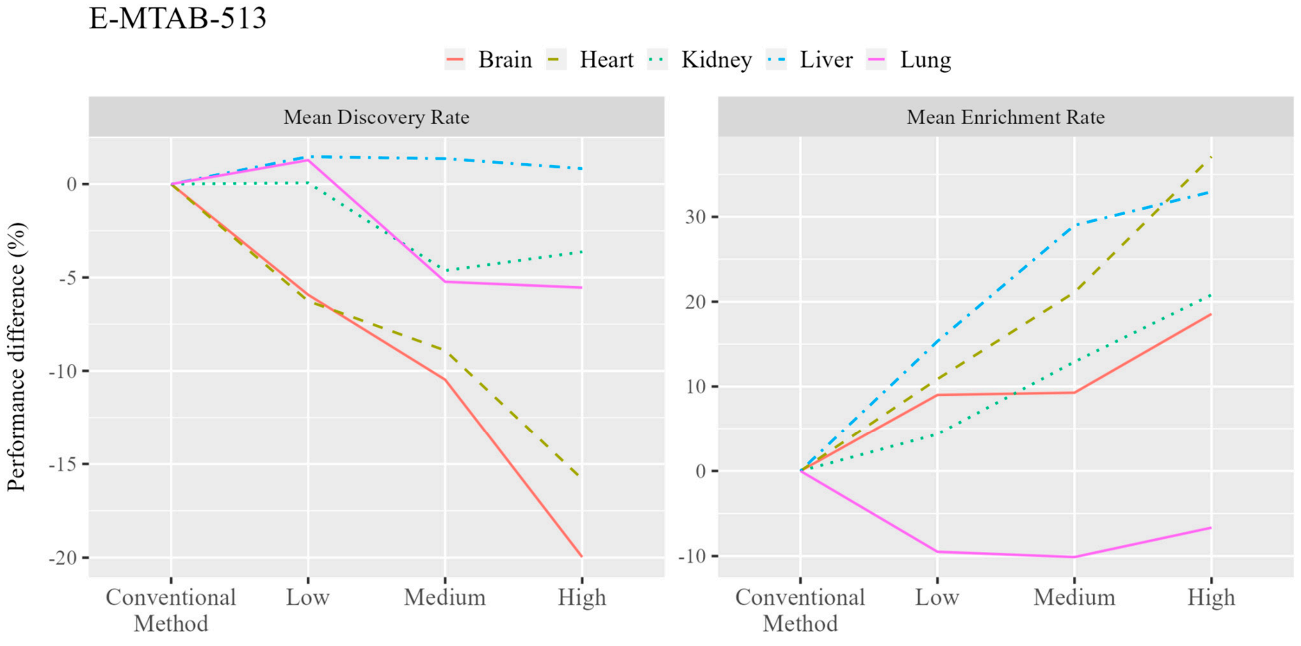

Figure S2. The performance of conventional disease inference and inference by incorporating tissue expression filters for the E-MTAB-513 dataset.

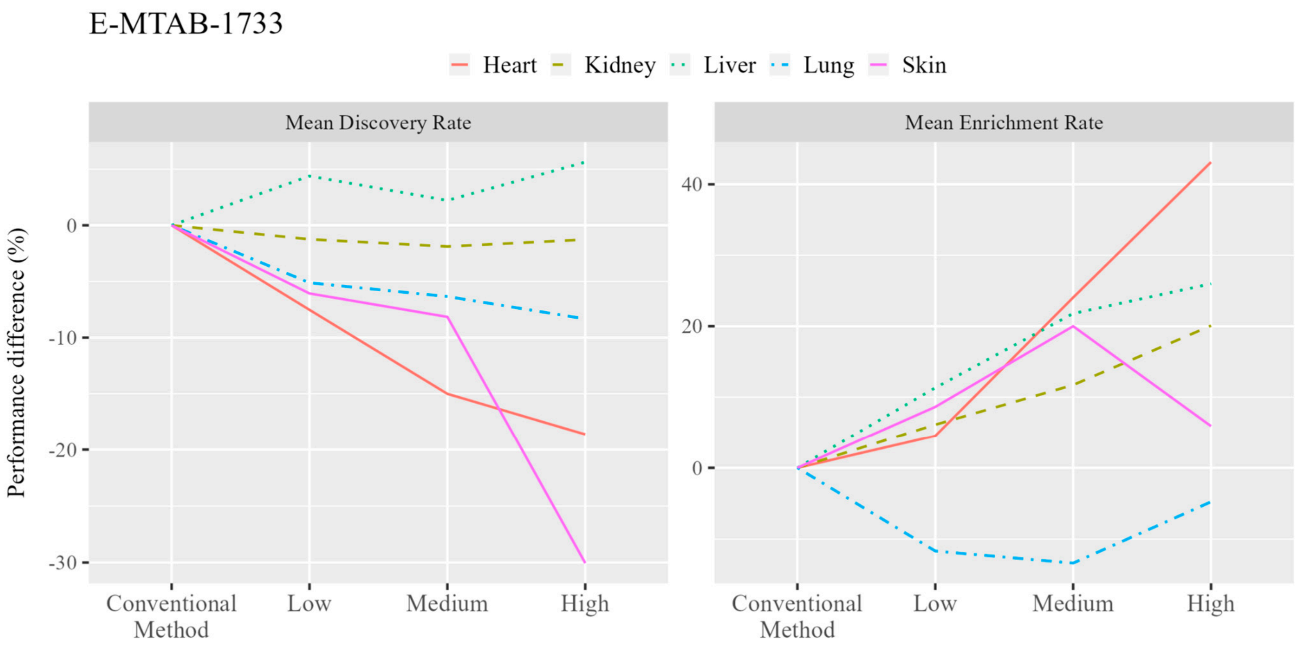

Figure S3. The performance of conventional disease inference and inference by incorporating tissue expression filters for the E-MTAB-1733 dataset.

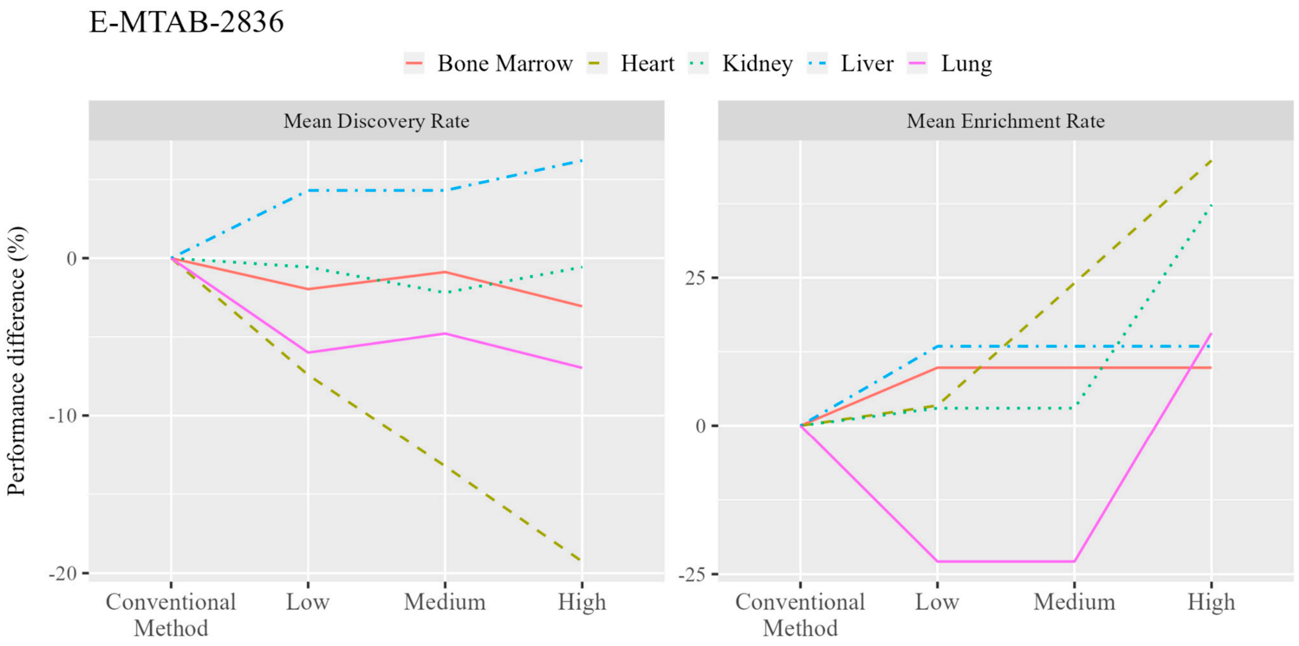

Figure S4. The performance of conventional disease inference and inference by incorporating tissue expression filters for the E-MTAB-2836 dataset.

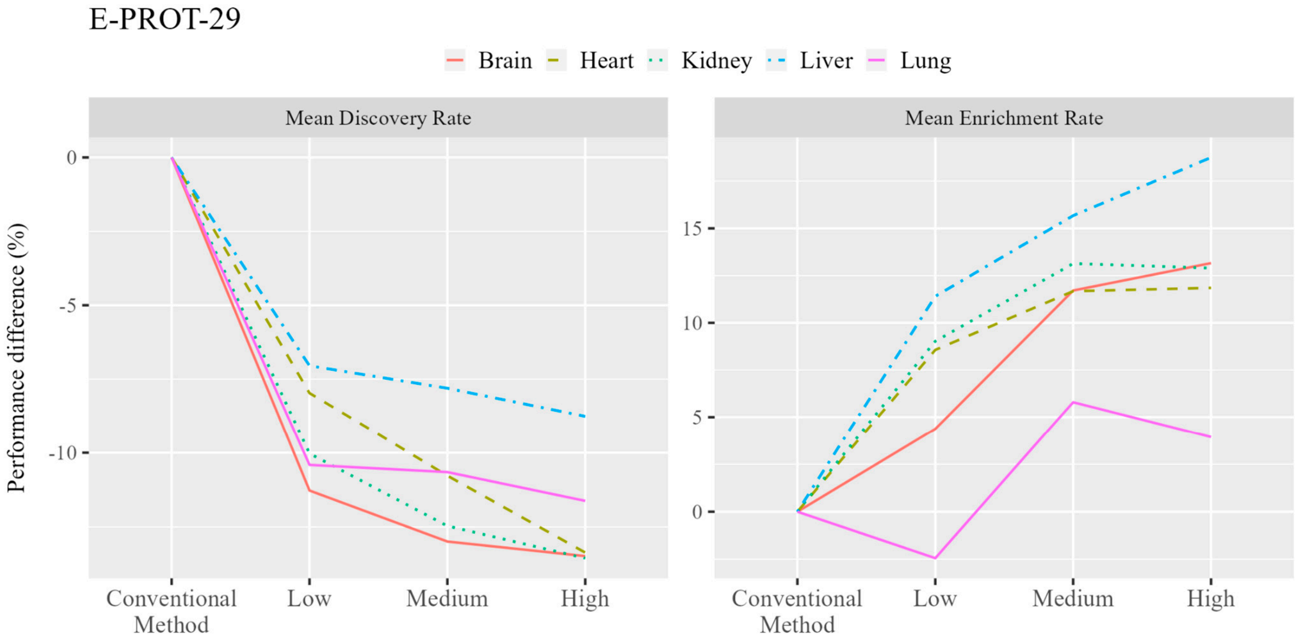

Figure S5. The performance of conventional disease inference and inference by incorporating tissue expression filters for the E-PROT-29 dataset.

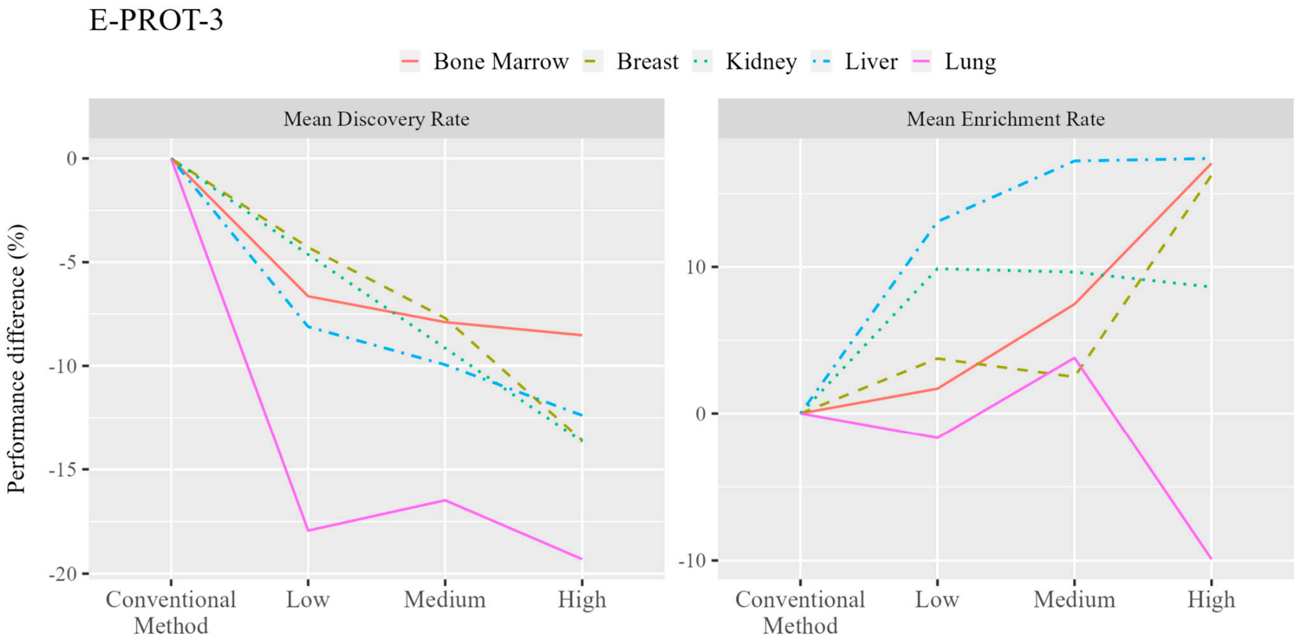

Figure S6. The performance of conventional disease inference and inference by incorporating tissue expression filters for the E-PROT-3 dataset.

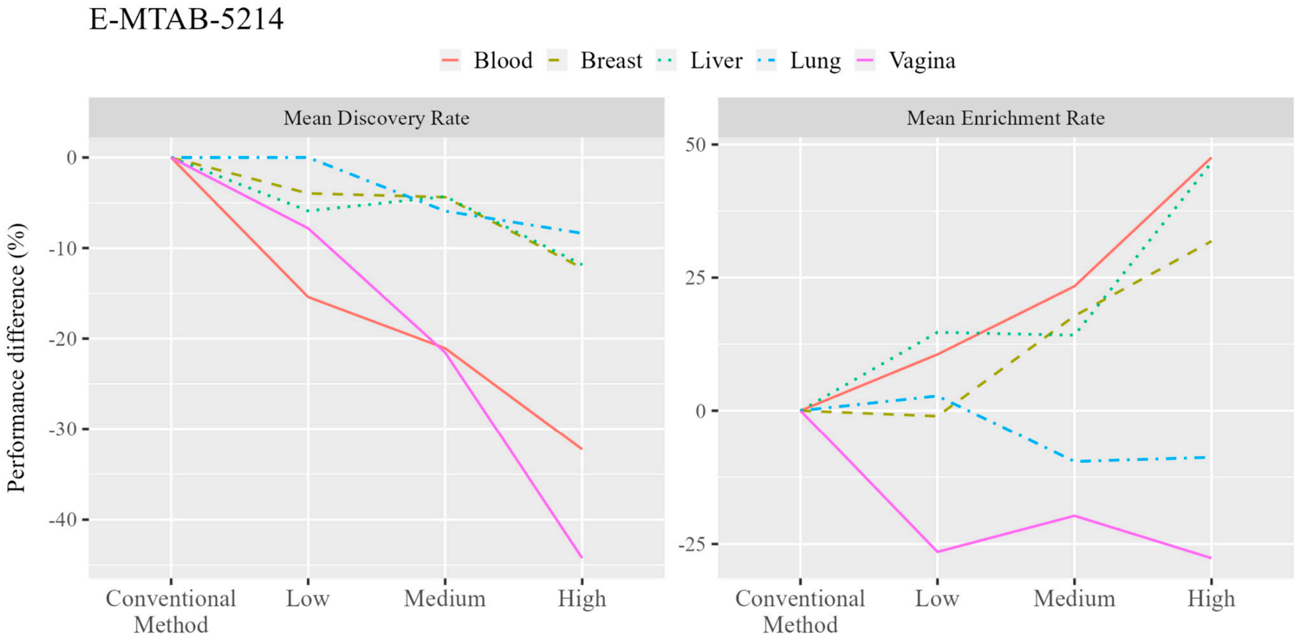

Figure S7. The performance for identifying disease-relevant chemicals of the conventional method and augmented methods by incorporating tissue expression filters for the E-MTAB-5214 dataset.

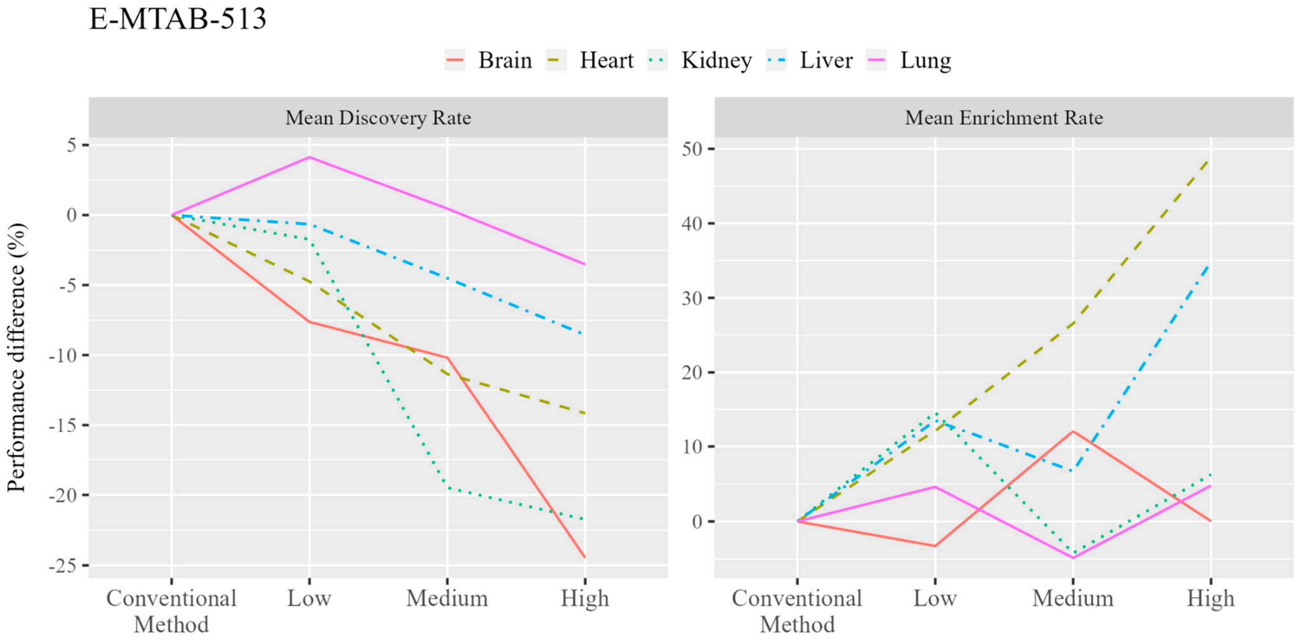

Figure S8. The performance for identifying disease-relevant chemicals of the conventional method and augmented methods by incorporating tissue expression filters for the E-MTAB-513 dataset.

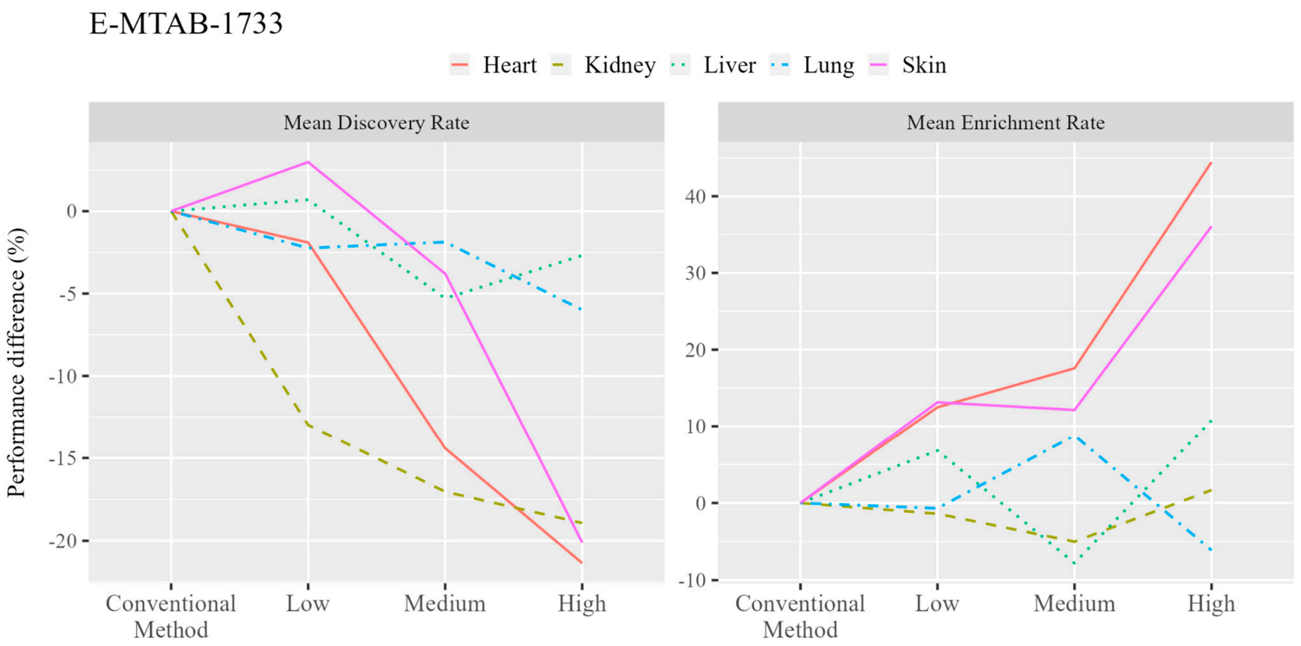

Figure S9. The performance for identifying disease-relevant chemicals of the conventional method and augmented methods by incorporating tissue expression filters for the E-MTAB-1733 dataset.

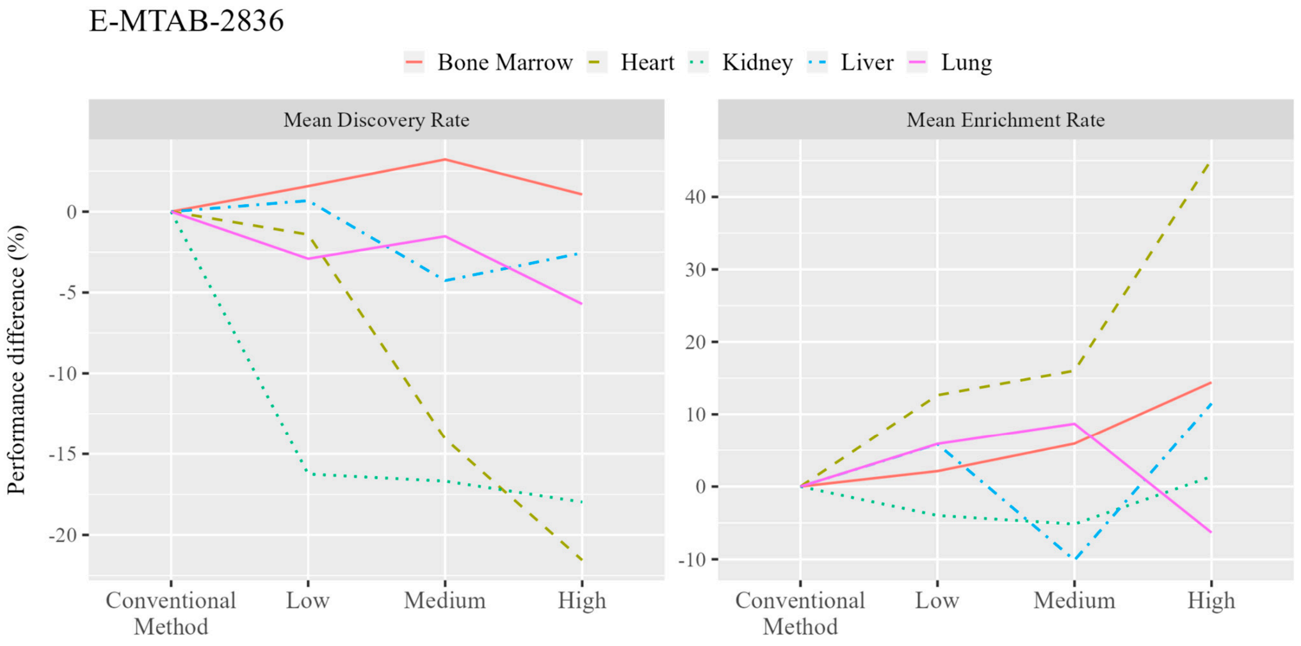

Figure S10. The performance for identifying disease-relevant chemicals of the conventional method and augmented methods by incorporating tissue expression filters for the E-MTAB-2836 dataset.

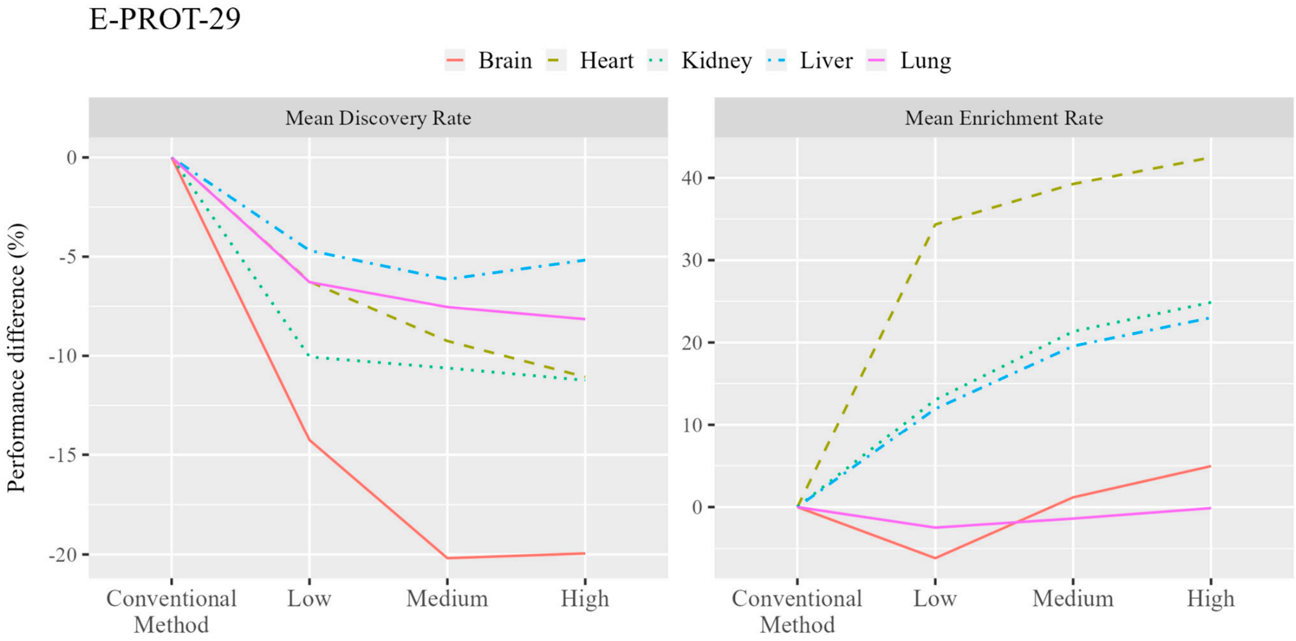

Figure S11. The performance for identifying disease-relevant chemicals of the conventional method and augmented methods by incorporating tissue expression filters for the E-PORT-29 dataset.

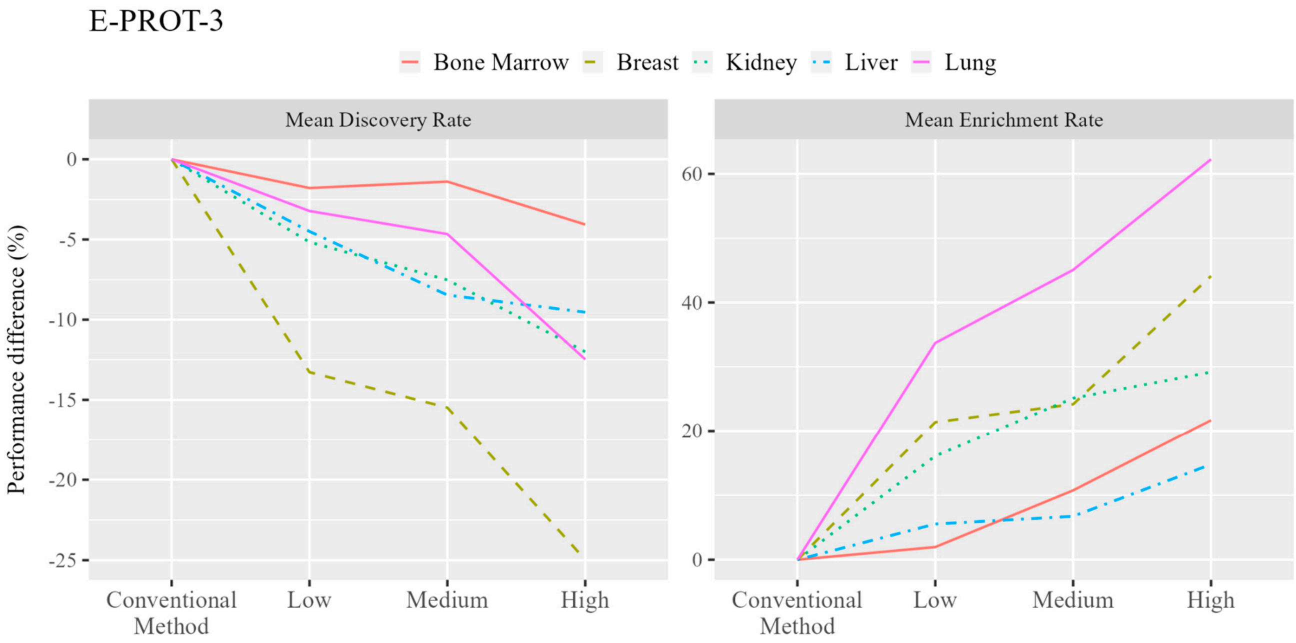

Figure S12. The performance for identifying disease-relevant chemicals of the conventional method and augmented methods by incorporating tissue expression filters for the E-PORT-3 dataset.
